# Anti-Photoaging Effects of Four Insect Extracts by Downregulating Matrix Metalloproteinase Expression via Mitogen-Activated Protein Kinase-Dependent Signaling

**DOI:** 10.3390/nu11051159

**Published:** 2019-05-23

**Authors:** A-Rang Im, Kon-Young Ji, InWha Park, Joo Young Lee, Ki Mo Kim, MinKyun Na, Sungwook Chae

**Affiliations:** 1 Herbal Medicine Research Division, Korea Institute of Oriental Medicine, Yuseong-daero 1672, Yuseong-gu, Daejeon 34054, Korea; lar744@hanmail.net (A.-R.I.); jky8387@kiom.re.kr (K.-Y.J.); jy0130@kiom.re.kr (J.Y.L.); vsrc@kiom.re.kr (K.M.K.); 2 College of Pharmacy, Chungnam National University, 99 Daehak-ro, Yuseong-gu, Daejeon 34134, Korea; inwha129@naver.com; 3 Korean Convergence Medicine, University of Science and Technology, 217 Gajeong-ro, Yuseong-gu, Daejeon 34113, Korea

**Keywords:** matrix metalloproteinase, ultraviolet B, insect extracts, transepidermal water loss, MAPKs, pro-inflammatory cytokines, photoaging

## Abstract

Insects are some of the most diverse organisms on the planet, and have potential value as food or medicine. Here, we investigated the photoprotective properties of insect extracts using hairless mice. The alleviating wrinkle formation effects of insect extracts were evaluated by histological skin analysis to determine epidermal thickness and identify collagen fiber damage. Moreover, we investigated the ability of the insect extracts to alleviate UVB-induced changes to matrix metalloproteinases (MMPs), oxidative damage, the mitogen-activated protein kinases (MAPKs) signaling pathway, and the expression of pro-inflammatory cytokines. Insect extracts reduced UVB-induced skin winkles, epidermal thickening, and collagen breakdown, and alleviated the epidermal barrier dysfunction induced by UVB, including the increased loss of transepidermal water. Moreover, the expression of skin hydration-related markers such as hyaluronic acid, transforming growth factor-beta (TGF-β), and procollagen was upregulated in the group treated with insect extracts compared to the vehicle-treated group after ultraviolet B (UVB) exposure. UVB irradiation also upregulated the expression of MMPs, the phosphorylation of MAPKs, and pro-inflammatory cytokines, which were all attenuated by the oral administration of insect extracts. These results indicate the photoaging protection effect of insect extracts and the underlying mechanism, demonstrating the potential for clinical development.

## 1. Introduction

Edible insects have long formed an integral component of the genetic and cultural heritage in different regions worldwide [1]. The Food and Agriculture Organization of the United Nations (FAO) has proposed edible insects to reduce environmental pollution and support nutritional supplementation, and has recently investigated the quality, safety, and nutrients of edible insects in a number of studies [2,3,4,5]. Since insects and their components also show potential as a new source of medicinal and medicinal ingredients, we have confirmed the protective effect of insect extracts against skin damage by ultraviolet light. 

Extrinsic aging factors during skin aging include exposure to sunlight, pollution, nicotine, and lifestyle components; among these factors, exposure to ultraviolet (UV) irradiation is related with a range of skin diseases [6,7]. UV-induced photoaging results in changes to the dermis, which is evaluated by induced roughness and dryness, wrinkle formation, irregular pigmentation, and the loss of elasticity [8].

The characteristics of skin barrier function above include an increase of transepidermal water loss and skin capacitance hydration; thus, the moisture loss of epidermis is an important physiological index to impaired skin barrier function [9]. Hyaluronic acid (HA) is the important component of the dermal and epidermal layer, and the decrease of HA caused by UV is important for maintaining skin health, because it reduces the skin’s elasticity and moisture content [10,11].

A molecular chain reaction in the dermis is stimulated by UV radiation, and ultimately results in the increased production of matrix metalloproteinases (MMPs) in dermis and epidermis, which upregulate the stimulation of collagenase production in fibroblasts and keratinocytes [12]. Excessive levels of MMPs by photoaging can induce the impairment of structural integrity in the dermis and epidermis, which is evaluated by increased roughness, thickening, coarse wrinkles, and irregular pigmentation [13]. In particular, the increased expression of MMP-1 due to an increased expression of mitogen-activated protein kinases (MAPKs) reduces skin elasticity, wrinkles, and inflammation by destroying the collagen matrix and skin tissue [14]. The induction of reactive oxygen species (ROS) by ultraviolet B (UVB) irradiation and subsequent inflammatory reactions induce the expression of pro-inflammatory cytokines such as interleukin (IL)-1β, IL-6, and tumor necrosis factor-alpha (TNF-α) in skin cells, including fibroblasts and keratinocytes [15].

Herein, we confirmed the photoprotective efficacy on UV-induced skin damage of extracts from four different insect species: *Allomyrina dichotoma* larva (ADL), *Protaetia brevitarsis seulensis* (PBS), *Tenebrio molitor* Linnaeus (TML), and *Gryllus bimaculatus* De Geer (GBD). These four edible insects have been registered in Food Standards and Specificaitons (No. 2017-102, 15 December 2017) of the Ministry of Food and Drug Safety of Korea (MFDS), and can be distributed and sold as food [16]. We prepared a 70% ethanol extract of the insects, which we investigated for potential skin protection effects against UVB-induced damage. Then, these effects were examined by various parameters of photoaging.

## 2. Materials and Methods

### 2.1. Preparation of Insects Extracts

Dried insects (ADL, PBS, TML, and GBD) were obtained from a commercial supplier, and all voucher specimens were deposited at the herbal bank of the Korea Institute of Oriental Medicine (Table 1). The dried insects were ground into fine powder and extracted with 70% ethanol. The 70% ethanol extracts were filtered and evaporated. Finally, all insect extracts were used after being lyophilized in a freeze dryer.

### 2.2. Experimental Animals and Oral Administration

HR-1 hairless male mice were used in this study, which were obtained from Japan SLC, Inc. (Shizuoka, Japan). The HR-1 hairless mice were stabilized for one week before the study. The mice were housed in specific pathogen-free conditions and a climate-controlled facility at 24 °C, 50% humidity, and under cycles of 12:12 h light and dark with free access to food and water. The animal experiments were approved by Institutional Animal Care and Use Committee of Korea Institute of Oriental Medicine (17-051). The mice were separated into six groups (n = 6 each): the normal control, ultraviolet B (UVB)-irradiated vehicle, UVB-irradiated ADL, UVB-irradiated PBS, UVB-irradiated TML, and UVB-irradiated GBD groups and 0.1 mL of extracts containing 100 mg/kg body weight was orally administered every day for 12 weeks. The normal control group was not exposed to UVB nor administered any extract, and the vehicle group was treated only with UVB.

### 2.3. UVB Irradiation 

UVB irradiation was performed using a UVM-225D Mineralight UV display lamp (UVP, Phoenix, AZ, USA) that emitted radiation at a wavelength of 302 nm. The UVB radiation was applied to the backs of the mice three times per week for 12 weeks and was progressively increased from 60 mJ/cm^2^ per exposure during week one (one minimal erythematous dose = 60 mJ/cm^2^) to 90 mJ/cm^2^ per exposure until week 12. The strength of the UV radiation was measured using an HD2102-2 UV meter (Delta OHM, Padova, Italy).

### 2.4. Skin Hydration and Transepidermal Water Loss (TEWL) 

TEWL and capacitance were measured after UVB radiation. A corneometer and a Tewameter (both Courage + Khazaka electronic GmbH, Cologne, Germany) were respectively used to measured skin hydration and TEWL, which is a marker of the barrier function of the epidermis. 

### 2.5. Histological Investigation

For histological investigation, the dorsal skin was removed from each hairless mouse. The histological investigation used a conventional method. Briefly, the skin tissues were fixed in 10% neutral-buffered formalin and washed with distilled water. The fixed tissues were dehydrated, cleaned, and then infiltrated with and embedded in paraffin wax. After being embedded, the fixed tissues were cut into 5-μm sections. The section of skin samples was performed with hematoxylin and eosin (H & E) and Masson’s trichrome staining to evaluate a collagen fiber organization. The analyzed epidermal thickness was the distance between the keratin layer and the epidermal basement membrane, which was measured by light microscopy using an eyepiece micrometer (Olympus Corporation, Tokyo, Japan). 

### 2.6. Determination of MMP-1, MMP-9, and Hyaluronic Acid (HA) Secretion Using Enzyme-Linked Immunosorbent Assay (ELISA) 

MMP-1, MMP-9, and HA levels in the skin tissue after UVB irradiation were analyzed by total MMP-1, MMP-9, and HA ELISA kits following the manufacturer’s protocol. The levels of MMP-1, MMP-9, and HA were measured using colorimetric analysis with a microplate reader (Molecular Devices, Sunnyvale, CA, USA).

### 2.7. Antioxidant Enzyme Activities

Any superoxide dismutase (SOD) and catalase (CAT) activity were analyzed by a colorimetric assay kit (Cayman Chemical Co., Ann Arbor, MI, USA) following the manufacturer’s instructions. For protein extraction, skin tissue samples were homogenized using cold lysis buffer, and the activity of SOD and CAT were measured at the absorbance at 450 nm and 540 nm, respectively, using a microplate reader (Molecular Devices).

### 2.8. RNA Extraction and Quantitative Real-Time Polymerase Chain Reaction (qRT-PCR)

The total RNA of the skin tissue from UVB-irradiated mice was extracted by TRIzol reagent (Invitrogen, Carlsbad, CA, USA) following the manufacturer’s protocol. Dried pellets were resuspended in 20 μL of diethyl pyrocarbonate (DEPC)-water. RNA concentration was determined using a NanoDrop instrument. To generate cDNA, purified RNA was synthesized by the High Capacity cDNA Reverse Transcription kit (Applied Biosystems), according to the manufacturer’s instructions. qRT-PCR was performed using TaqMan assays (Applied Biosystems, Foster City, CA, USA) with primers specific for transforming growth factor-beta (TGF-β), Mm00436960_m1; IL-1β, Mm00434228_m1; IL-6, Mm00446190_m1; and TNF-α, Mm00443258_m1 using a QuantStudio^TM^ 6 Flex real-time PCR system (Applied Biosystems). Each sample was assayed in triplicate, and the relative mRNA expression levels in each sample were calculated using the ΔΔcycle threshold (Ct) method and normalized to the β-actin mRNA levels [17]. 

### 2.9. Western Blotting

Protein was extracted from the skin tissue of UVB-irradiated hairless mice. An equal concentration of protein samples (20 µg) were separated on a 10% sodium dodecyl sulfate-polyacrylamide gel and transferred to polyvinylidene fluoride (PVDF) membranes. After transfer, the PVDF membranes were blocked for 1 h at room temperature with a 5% blocking solution (ATTO, Tokyo, Japan). After blocking, they were incubated at 4 °C overnight with indicated primary antibodies (MMP-1 (#54376), MMP-9 (#13667), pERK (#9101), ERK (#9102), pMEK (#9154), MEK (#9126), pp38 (#9215), p38 (#9212), pJNK (#9251), JNK (#9252), and β-actin (#4970) were purchased from Cell signaling, diluted (1:1000), washed three times for 10 min each in Tris-buffered saline (TBS), and then incubated for 2 h at room temperature with the anti-rabbit secondary antibody (#7074, Cell signaling). The proteins were developed by an enhanced chemiluminescence (ECL) solution and detected with a LAS-4000 mini luminescent image analyzer (GE Heathcare, UK).

### 2.10. Statistical Analysis 

All the measurements were performed in triplicate, and the values are presented as the mean ± standard errors. An analysis of variance using Tukey’s test was used to analyze the differences in the results among the study groups, and *p* < 0.05 was considered significant.

## 3. Results

### 3.1. Effects of Insect Extracts on Skin Hydration Factors in UVB-Irradiated Hairless Mice

**Figure 1:**
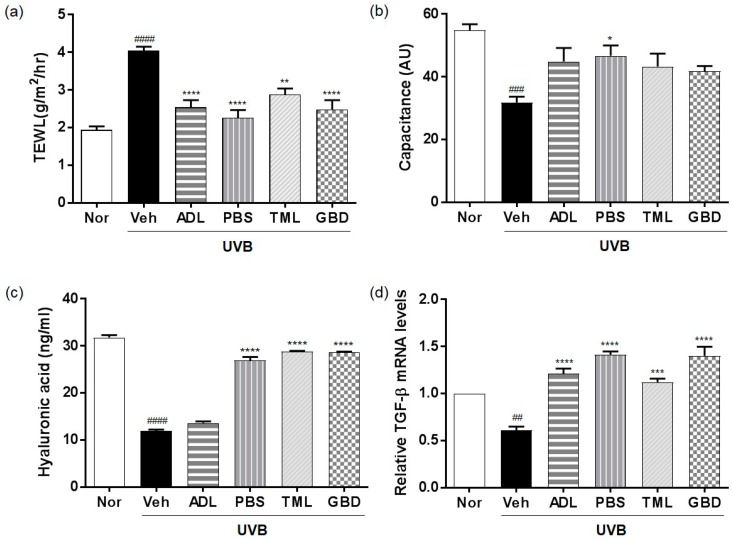
Effects of insect extracts on ultraviolet B (UVB)-induced skin hydration. (**a**) Transepidermal water loss (TEWL) and (**b**) capacitance in UVB-irradiated hairless mice after the administration of insect extracts for over 10 weeks. (**c**) Hyaluronic acid (HA), and (**d**) transforming growth factor-beta (TGF-β) mRNA. #### *p* < 0.0001, ### *p* < 0.001 and ## *p* < 0.01 vs. the normal group, and **** *p* < 0.0001, *** *p* < 0.001, ** *p* < 0.01 and * *p* < 0.05 vs. the vehicle group. These measurements were performed in triplicate. Nor, Normal; Veh, Vehicle; ADL, *Allomyrina dichotoma* larva; PBS, *Protaetia brevitarsis seulensis*; TML, *Tenebrio molitor* Linnaeus; GBD, *Gryllus bimaculatus* De Geer.

The TEWL tended to be much higher in the UVB-treated vehicle group (Figure 1a), but was lower in the UVB-treated insect extracts groups compared to that of the vehicle group. The capacitance was reduced in the UVB-treated vehicle group, but increased in the UVB-treated insect extracts groups (Figure 1b). In addition, the HA content in skin and TGF-β mRNA levels were higher in the UVB-exposed insect extract-treated group than in the UVB-exposed vehicle group (Figure 1c,d). 

### 3.2. Effect of Insect Extracts on Restoration of Skin Thickening and Collagen Destruction on UVB-Irradiated Hairless Mice

**Figure 2:**
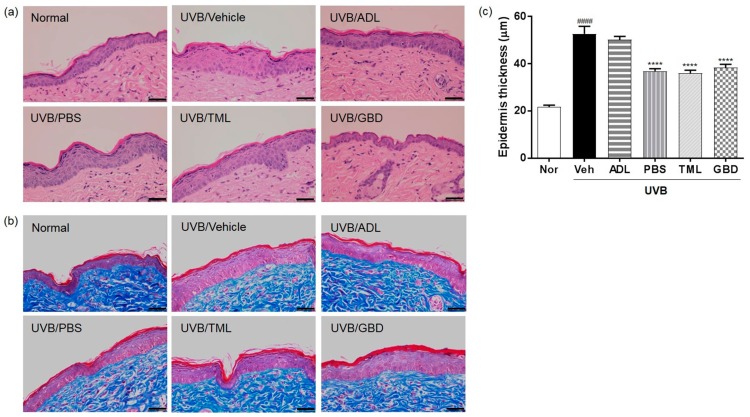
Effect of insect extracts on UVB-induced skin thickening in hairless mice. (**a**) Hematoxylin and eosin (H & E) staining of UVB-irradiated hairless mouse skin. (**b**) Protective effect of insect extracts on changes in collagen fibers. Histological observation of hairless mouse skin using Masson’s trichrome staining. Collagen fibers are stained blue, and images were obtained at ×400 magnification. The image data were represented individual mice (n = 6 per group). (**c**) Dorsal skin epidermal thickness. #### *p* < 0.0001 vs. the normal group, and **** *p* < 0.0001, vs. the vehicle group. These measurements were performed in triplicate. Scale bar; black bold line: 50 µm. Nor, Normal; Veh, Vehicle; ADL, *Allomyrina dichotoma* larva; PBS, *Protaetia brevitarsis seulensis*; TML, *Tenebrio molitor* Linnaeus; GBD, *Gryllus bimaculatus* De Geer.

To investigate the anti-wrinkle effects of the insect extracts, tissue sections from the skin of hairless mice were stained with H & E and Masson’s trichrome. The depths of the stratum corneum and epidermis were markedly increased in the skin of the UVB-irradiated vehicle group compared to the normal group, and were restored by the treatment of insect extracts (Figure 2a). The uniformly distributed collagen was observed in the dermal layer by Masson’s trichrome staining, and the collagen fibers were restored in the UVB-irradiated insect extracts group compared with the vehicle group (Figure 2b). To further evaluate the anti-wrinkle effects of the insect extracts, we measured the distance between the keratin layer and the epidermal basement membrane using microscopic images of H & E-stained skin sections. As shown in Figure 2c, the epidermal thickness of the skin was markedly increased in the UVB-irradiated vehicle group compared to the normal group. Moreover, the increased epidermal thickness of skin was reduced by the treatment of insect extracts.

### 3.3. Insect Extracts Inhibit UVB-Induced MMP-1 and MMP-9 Expression

**Figure 3:**
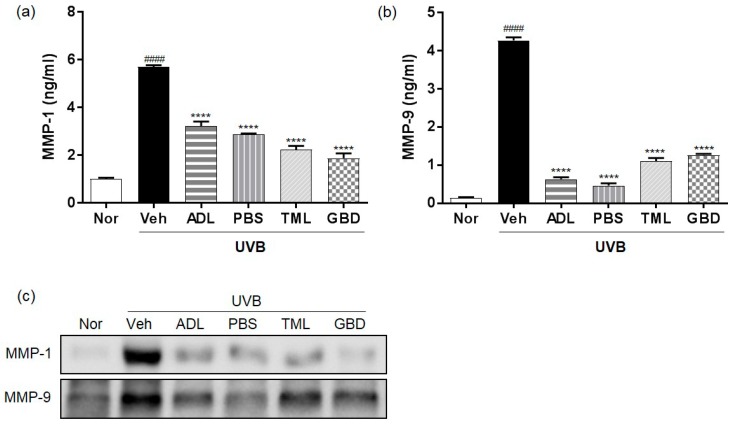
Effect of insect extracts on matrix metalloproteinase (MMP) and procollagen expression. (**a**) MMP-1 and (**b**) MMP-9 protein levels in UVB-irradiated skin. (**c**) Western blot analysis of effects of insect extracts on UVB-mediated induction of MMP-1 and MMP-9. #### *p* < 0.0001 vs. normal group, **** *p* < 0.0001 vs. vehicle group. These measurements were performed in triplicate. Nor, Normal; Veh, Vehicle; ADL, *Allomyrina dichotoma* larva; PBS, *Protaetia brevitarsis seulensis*; TML, *Tenebrio molitor* Linnaeus; GBD, *Gryllus bimaculatus* De Geer.

To evaluate the regulation of MMPs’ expression by insect extracts, we analyzed the protein levels of MMPs using ELISA and Western blotting. The protein level of MMP-1 and MMP-9 was upregulated in the skin of the UVB-irradiated vehicle group compared to normal group, and it was downregulated by the treatment of insect extracts (Figure 3a,b). Consistently, Western blotting also showed that the protein expression of MMP-1 and MMP-9 was induced by UVB irradiation, and it was suppressed by the treatment of insect extracts (Figure 3c).

### 3.4. Effects of Insect Extracts on Antioxidant Enzymes in UVB-Irradiated Hairless Mice

**Figure 4:**
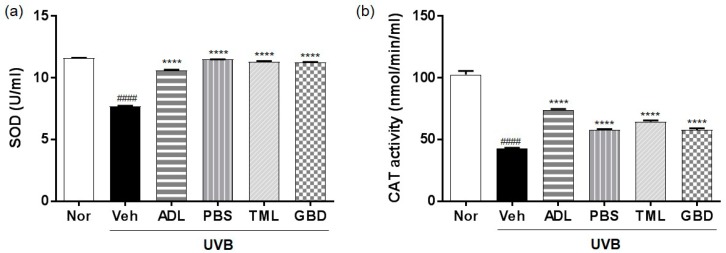
Effects of insect extracts on antioxidant activity with respect to (**a**) superoxide dismutase (SOD) and (**b**) catalase (CAT) in hairless mice skin exposed to UVB. #### *p* < 0.0001 vs. normal group, **** *p* < 0.0001 vs. vehicle group. These measurements were performed in triplicate. Nor, Normal; Veh, Vehicle; ADL, *Allomyrina dichotoma* larva; PBS, *Protaetia brevitarsis seulensis*; TML, *Tenebrio molitor* Linnaeus; GBD, *Gryllus bimaculatus* De Geer.

To determine the free radical-scavenging effect of insect extracts due to the regulation of antioxidant enzymes, the activity of SOD and CAT was measured in the skin of UVB-irradiated hairless mice. The activity of SOD was suppressed in the UVB-irradiated vehicle group compared to the normal group (Figure 4a), and it was restored by insect extract treatment. Furthermore, the activity of CAT was also suppressed by UVB irradiation, and it was recovered by the oral administration of insect extracts (Figure 4b). 

### 3.5. Effects of Insect Extracts on MAPK Phosphorylation in UVB-Irradiated Hairless Mice

**Figure 5:**
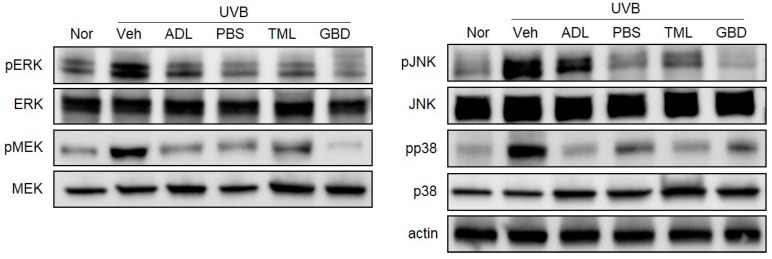
Effects of insect extracts on the phosphorylation of mitogen-activated protein kinases (MAPKs) in the UVB-irradiated mouse model. Insect extracts inhibited the phosphorylation of MAPK kinase (MEK), extracellular signal-regulated kinase (ERK), p38, and c-Jun N-terminal kinase (JNK). The prefix p means phosphorylation. Nor, Normal; Veh, Vehicle; ADL, *Allomyrina dichotoma* larva; PBS, *Protaetia brevitarsis seulensis*; TML, *Tenebrio molitor* Linnaeus; GBD, *Gryllus bimaculatus* De Geer.

It is well-known that the three subfamilies of the MAPKs pathway are extracellular signal-regulated kinase (ERK), c-Jun N-terminal kinase (JNK), and p38. To determine the regulation of the MAPKs pathways by the treatment of insect extracts, we analyzed the phosphorylation of MEK, ERK, JNK, and p38 by Western blotting. As shown in Figure 5, UVB irradiation induced the phosphorylation of MEK, ERK, JNK, and p38 in hairless mouse skin, while their total protein expressions showed no difference. In addition, the UVB-induced phosphorylation of MEK, ERK, JNK, and p38 was attenuated by the pretreatment of insect extracts.

### 3.6. Effects of Insect Extracts on the mRNA Expression of Inflammatory Cytokines in UVB-Irradiated Hairless Mice

**Figure 6:**
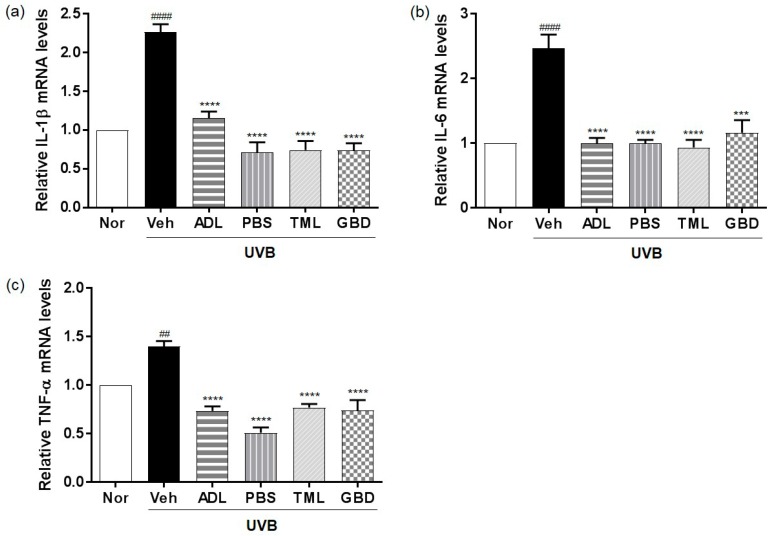
Effects of insect extracts on pro-inflammatory cytokines in UVB-irradiated hairless mouse skin. mRNA expression levels of (**a**) interleukin (IL)-1β, (**b**) IL-6, and (**c**) tumor necrosis factor-alpha (TNF-α) were determined by quantitative reverse transcription–polymerase chain reaction. #### *p* < 0.0001 and ## *p* < 0.01 vs. normal group, **** *p* < 0.0001, and *** *p* < 0.001 vs. vehicle group. These measurements were performed in triplicate. Nor, Normal; Veh, Vehicle; ADL, *Allomyrina dichotoma* larva; PBS, *Protaetia brevitarsis seulensis*; TML, *Tenebrio molitor* Linnaeus; GBD, *Gryllus bimaculatus* De Geer.

The qRT-PCR results further confirmed that the mRNA levels of the pro-inflammatory cytokines including IL-1β, IL-6, and TNF-α were increased by UVB exposure, and were significantly downregulated by the insect extracts (Figure 6). 

## 4. Discussion

Extrinsic aging is considered to be the most direct cause of photoaging; the skin becomes dry, rough, and thick, resulting in wrinkles and the loss of skin elasticity [18]. The most notable features of skin by UV radiation are an inordinate deposition of abnormal elastin complex and the impairment of collagen fibers, which are accompanied by skin thickening, depigmentation, and telangiectasia; so, protection from UV light is important [19,20]. 

Edible insects are known to be a source of potential food and raw material for medicine, and further research is needed on their bioactive principles. However, some recent studies reported that nutrients and secondary metabolites are associated with this effect. These effects are related to the nutritional value such as the vitamins, minerals, fatty acids, and amino acids, as well as the secondary metabolites including indole alkaloids and nitrogenous compounds [3,16,21,22]. In this study, 70% ethanol extract was used instead of whole insects, which might suggest that short-chain fatty acids, peptides, and secondary metabolites, etc. might be involved in their biological function rather than primary metabolites such as protein, fat, and amino acids. A systematic study on the isolation and structural determination of components explaining the pharmacological mechanisms remains for further research.

The protective effects on skin using various insect extracts prepared from ADL, PBS, TML, and GBD were evaluated, which are all considered as potential food sources of the future. ADL is widely administered in traditional medicine, and has a therapeutic potential for anti-diabetes, anti-obese, anti-cancer, and anti-hepatic fibrosis [23,24]. PBS is temporarily admitted by the Ministry of Food and Drug Safety of Korea (MFDS) as a food material, and shows inhibitory effects against platelet aggregation and thrombosis [22].

Skin hydration is strongly linked to skin barrier function, and is important for maintaining a healthy skin barrier [25]. UV irradiation increases the TEWL to result in the damage or weakening of the skin barrier function, leading to wrinkle formation [26]. Also, HA and TGF-β are well-known to improve skin hydration reduced by UVB irradiation [27]. We confirmed that UVB irradiation exposure to mice induced TEWL and decreased capacitance, whereas the oral administration of insect extracts impeded this UV-induced damage to skin barrier function. In addition, the insect extracts prevented the UVB-induced the inhibition of HA, TGF-β, and procollagen expression.

Recent studies have reported that the prominent features of photodamaged skin are the induction of epidermal thickness and changes of connective tissue organization using histological and ultrastructural studies of skin [28]. In addition, UV-induced MMPs, as a major marker of photoaging and skin inflammation, excessively degraded a collagen and extracellular matrix (ECM) [29]. MMPs play an important role in several normal and abnormal physiological processes such as the degradation of the ECM, including collagens and elastin [30,31]. Consistently, our data illustrated that the treatment of insect extracts maintained the collagen levels and restored the epidermal thickness of skin from UVB-irradiated hairless mice, and the increased expression of MMPs was reduced by the treatment of insect extracts. These results implied that insect extracts have protective effects against wrinkle formation via collagen degradation, which is accompanied by the increased expression of MMPs. 

The human skin has a network of protective antioxidants such as the enzymatic antioxidants SOD and CAT [32], and we found that the insect extracts could increase the levels of these enzymes to protect against the ROS generation induced by UVB exposure. Our results further showed that the insect extracts mediate their photoprotective effects by downregulating the UVB-induced activation of the MAPKs signaling pathway, including ERK, p38, and JNK [33], which are inflammatory mediators. UV-induced inflammatory changes manifest as an accumulation of the pro-inflammatory cytokines including IL-1, IL-6, and TNFα [34]. We found the suppressed expression of pro-inflammatory cytokines, suggesting the inhibitory effect of insect extracts on the inflammatory mediator production. 

In conclusion, there is a clear photoprotective effect of insect extracts against UVB-induced skin damage in hairless mice. Insect extracts inhibited skin thickening and wrinkle formation, and increased skin hydration factors and antioxidant enzymes in hairless mice. Furthermore, insect extracts attenuated UVB-induced MMPs, pro-inflammatory cytokines, and MAPK phosphorylation. Overall, these results suggest that insect extracts could effectively prevent against UVB-induced skin damage.

## Figures and Tables

**Table 1:** The systematic family and order names of insects.

Scientific Name	Family Name	Order Name
*Allomyrina dichotoma* larva (ADL)	Scarabaeidae	Coleoptera
*Protaetia brevitarsis seulensis* (PBS)	Scarabaeidae	Coleoptera
*Tenebrio molitor* Linnaeus (TML)	Tenebrionidae	Coleoptera
*Gryllus bimaculatus* De Geer (GBD)	Gryllidae	Orthoptera
